# Engineering the glyoxylate cycle for chemical bioproduction

**DOI:** 10.3389/fbioe.2022.1066651

**Published:** 2022-12-02

**Authors:** Peng Yang, Wenjing Liu, Yanan Chen, An-Dong Gong

**Affiliations:** College of Life Science, Xinyang Normal University, Xinyang, China

**Keywords:** glyoxylate cycle, metabolic engineering, biosynthesis, TCA cycle, organic acids, amino acids, fatty acids

## Abstract

With growing concerns about environmental issues and sustainable economy, bioproduction of chemicals utilizing microbial cell factories provides an eco-friendly alternative to current petro-based processes. Creating high-performance strains (with high titer, yield, and productivity) through metabolic engineering strategies is critical for cost-competitive production. Commonly, it is inevitable to fine-tuning or rewire the endogenous or heterologous pathways in such processes. As an important pathway involved in the synthesis of many kinds of chemicals, the potential of the glyoxylate cycle in metabolic engineering has been studied extensively these years. Here, we review the metabolic regulation of the glyoxylate cycle and summarize recent achievements in microbial production of chemicals through tuning of the glyoxylate cycle, with a focus on studies implemented in model microorganisms. Also, future prospects for bioproduction of glyoxylate cycle-related chemicals are discussed.

## Introduction

**FIGURE 1:**
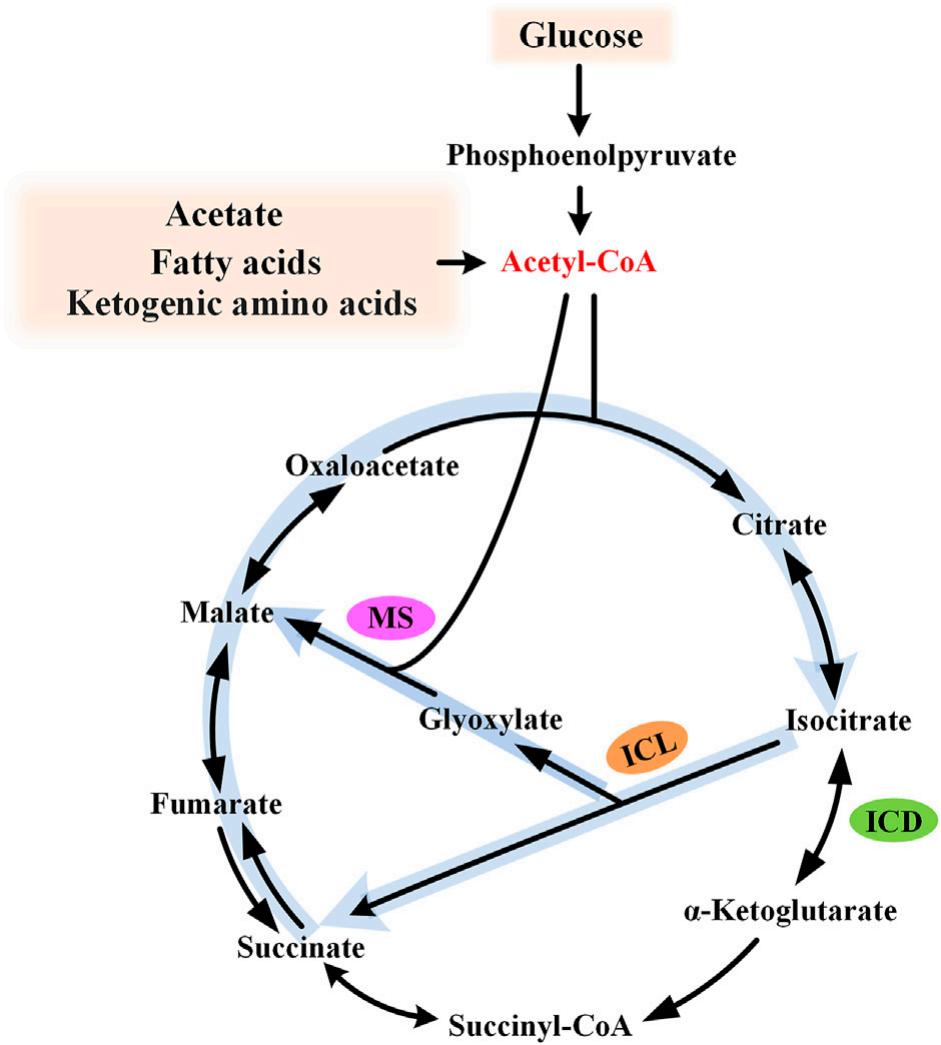
Diagram of the glyoxylate cycle. The glyoxylate cycle is shown by thick light blue arrows and the key intermediate acetyl-CoA is shown in red. ICL, isocitrate lyase; MS, malate synthase; and ICD, isocitrate dehydrogenase.

The glyoxylate cycle, also known as glyoxylate shunt (GS), was identified by Kornberg and Krebs in 1957, explaining how organisms could grow on acetate as the sole carbon source (Kornberg and Krebs, 1957). For substrates degraded exclusively to acetyl moieties (e.g., acetate, fatty acids, and ketogenic amino acids), this pathway provides a simple and efficient strategy for anaplerosis and gluconeogenesis, and, thus, cell growth. The glyoxylate cycle is generally regarded as an ancillary pathway of the TCA cycle, which is widely acknowledged as the central metabolic hub of the cell. This pathway comprises two dedicated enzymes: isocitrate lyase (ICL) and malate synthase (MS). ICL catalyzes the aldol cleavage of isocitrate to succinate and glyoxylate, while MS catalyzes the synthesis of malate from glyoxylate and acetyl-CoA. The overall effect of this pathway is the formation of one malate from two molecules of acetyl-CoA (Figure 1). It bypasses the oxidative decarboxylation steps of the TCA cycle and conserves carbon skeletons for biomass generation.

In addition to carbon assimilation, the glyoxylate cycle is also implicated in pathogenesis, antibiotic resistance, and oxidative stress tolerance. In view of its significance in metabolism and pathogenicity, the glyoxylate cycle has been extensively studied concerning the enzymology and metabolic regulation, particularly for *Escherichia coli* and the pathogenic bacterium *Mycobacterium tuberculosis* (Dolan and Welch, 2018). Undoubtedly, the gained knowledge lays a foundation for the bioproduction of related chemicals in metabolic engineering.

The glyoxylate cycle is involved in the synthesis of various chemicals. In recent years, studies on biosynthesis of organic acids, amino acids, and fatty acid-related products through GS engineering have been reported. Some of the outstanding results are presented in Table 1. To balance product output, reducing power regeneration, and cell growth, the glyoxylate cycle needs to be reinforced, weakened, fine-tuned, or dynamically controlled in different production cases. When heterologously expressed in some strains lacking this pathway, the GS amplified carbon source spectrum and enabled more metabolic flexibility, thus facilitating bioproduction (Kabisch et al., 2013; Schada von Borzyskowski et al., 2018; Shimizu et al., 2019).

**TABLE 1:** Representative studies of chemical biosynthesis through GS engineering.

Strain	Product	Substrate	Production capacity	Reference
*E. coli* Δ*adhE* Δ*ldhA* Δ*ack-pta* Δ*iclR*/pHL413	Succinate	Glucose	40 g/L in 96 h with a yield of 1.6 mol/mol	Sánchez et al. (2005)
*E. coli* Δ*ackA-pta* Δ*poxB* Δ*ldhA* Δ*adhE* Δ*mgsA* Δ*pflB* Δ*iclR* P_L_-*aceEF*-*lpdA*/pPYC	Succinate	Glucose	1.69 mol/mol in test-tube; 67.4 g/L in 26 h with a yield of 1.47 mol/mol during high cell density fermentation	Skorokhodova et al. (2015)
*E. coli* Δ*sdhAB* Δ*iclR* Δ*maeB*/pTrc99a-*gltA*	Succinate	Acetate	1.73 g/L in 72 h with a yield of 0.46 mol/mol; 7.29 g/L using resting-cells	Li Y et al. (2016)
*Methylomonas* sp. DH-1 Δ*sdh aceBA*	Succinate	Methane	195 mg/L with a yield of 0.0789 g/g	Nguyen et al. (2019)
*E. coli* BL21 (DE3) Δ*fumB* Δ*fumAC* Δ*aspA aceBAK:trc ppc:trc*/pSCppc	Fumarate	Glycerol	41.5 g/L in 82 h with a yield of 0.44 g/g	Li et al. (2014)
*E. coli* W3110 Δ*ldhA* Δ*poxB* Δ*pflB* Δ*pta-ackA* Δ*frdBC* Δ*fumB* Δ*fumAC*/pETM6R1-_(RBS10)_ *Af*PYC-*Ec*CS-*Ec*ACN-_(RBS10)_ *Ec*ICL-*Ec*SDH-dcuC	Fumarate	Glucose	22.4 g/L in 60 h	Chen et al. (2020)
*E. coli* B0013 Δ*adhE* Δ*ackA-pta* Δ*ldhA* Δ*maeA* Δ*maeB* Δ*mdh* Δ*iclR* Δ*arcA*/PETM7-PGNAB, sgRNA set 2, dCas9	Malate	Glucose	31.8 g/L in 50 h with a yield of 0.74 mol/mol	Gao et al. (2018)
*Aspergillus oryzae* C4T318 PC MDH ROPYC CS IDH OGD ACN ICL MS Sfc1p LlNOX RNAi-*cis*	Malate	Corn starch	117.2 g/L with a yield of 0.9 g/g and a productivity of 1.17 g/L/h	Liu et al. (2018)
*E. coli* W Δ*iclR*/pCDF_CAD, pET_ACS, pCOG5	Itaconate	Acetate	3.57 g/L in 88 h with a yield of 0.09 g/g	Noh et al. (2018)
*E. coli* MG1655 (DE3) Δ*ldhA* Δ*glcB* Δ*aceB* Δ*aldA*/pJNU-3, pJNU-4	Glycolate	Glucose	65.5 g/L in 77 h with a yield of 0.765 g/g	Deng et al. (2018)
*E. coli* Δ*xylB* Δ*glcD* Δ*aceB* Δ*glcB* Δ*gcl*/pGAx1, pGAx2	Glycolate	Xylose	40 g/L in 80 h with a yield of 0.63 g/g	Pereira et al. (2016)
*E. coli* Δ*aceB* Δ*gcl* Δ*glcDEFGB* Δ*iclR* Δ*edd-eda* Δ*arcA* Δ*icd* Δ*xylB khkC aldoB aldA ghrA aceA galP*	Glycolate	Glucose/xylose	3.73 g/L with a yield of 0.63 g/g	Alkim et al. (2016)
*E. coli* TWF001 Δ*iclR* Δ*lacI* Δ*fadR* Δ*fabR* Δ*lacA P*_*aceBA*_ *::P*_*trc*_ *P*_*aspC*_ *::P*_*trc*_ *P*_*acs*_ *::P*_*tac-trc*_ *P*_*aceB*_ *-aceBA P*_*fadB*_ *-fadBA P*_*tac*_ *-ppnK P*_*tac*_ -thrA*BC-rhtC P_*tac*_ *-aspC P*_*tac*_ *-ppc*	L-threonine	Glucose	103.89 g/l in 48 h with a yield of 0.72 g/g	Yang et al. (2019)
*E. coli* Δ*lacI* Δ*gabT* Δ*sucA*/pTA216, pTA1410, pTA1756	GABA	Glucose	4.8 g/Lwith a yield of 0.492 mol/mol and a productivity of 0.15 g/L/h	Soma et al. (2017)
*E. coli* Δ*ldhA* Δ*sdhA* Δ*iclR*/pK-hemA, pgRNA-L4, pdcas9-bacteria	5-Aminolevulinate	Glycerol	6.93 g/L in 18 h	Miscevic et al. (2021a)
*Corynebacterium glutamicum* Δ*aceB icd* ^*GTG*^/pVWEx1-*dpkA*_RBS^opt^, pEC-XT99A-*xylA*_*Xc*_ *-xylB*_*Cg*_	Sarcosine	Xylose/acetate	8.7 g/L with a yield of 0.25 g/g	Mindt et al. (2019a)
*E. coli* JCL16 Δ*ldhA* Δ*adhE* Δ*frdBC* Δ*pta*/pCS138, pTO1, pIM8	1-Butanol	Glucose	18.3 g/L in 78 h	Ohtake et al. (2017)
*E. coli* BL21 (DE3) Δ*poxB* Δ*adhE* Δ*ldhA* Δ*iclR*	3-Hydroxypropionate	Acetate	7.3 g/L with a yield of 0.26 mol/mol	Lama et al. (2021)
*Pseudomonas denitrificans* Δ*3hpdh* Δ*3hibdhIV* Δ*3hibdhI* Δ*pta-ackA* Δ*fabF*/pUCPK-AM	3-Hydroxypropionate	Acetate	3.64 g/L in 22 h	Zhou et al. (2020)
*E. coli* Δ*gabD* Δ*yneI* Δ*ldhA* Δ*adhE* Δ*pflB* Δ*ptsG*/p99S4CD, p15PGH	4-Hydroxybutyrate	Glycerol	103.4 g/L with a yield of 0.419 g/g and a productivity of 0.844 g/L/h	Choi S. Y et al. (2016)

In this review, we will briefly introduce the flux control of glyoxylate cycle in several different bacteria and recent achievements in biosynthesis of related chemicals. In addition, perspective for further study with the GS pathway will be presented.

## Regulation of the glyoxylate cycle

### The canonical regulation model in *E. coli*

A carbon flux is partitioned between the oxiditive TCA branch and GS at the isocitrate node, thereby, maintaining a balance of energy, reducing power, and gluconeogenic precursor production. In addition, glyoxylate is highly reactive or even toxic (due to the aldehyde group). Thus, tight regulation of the glyoxylate cycle is necessary.

**FIGURE 2:**
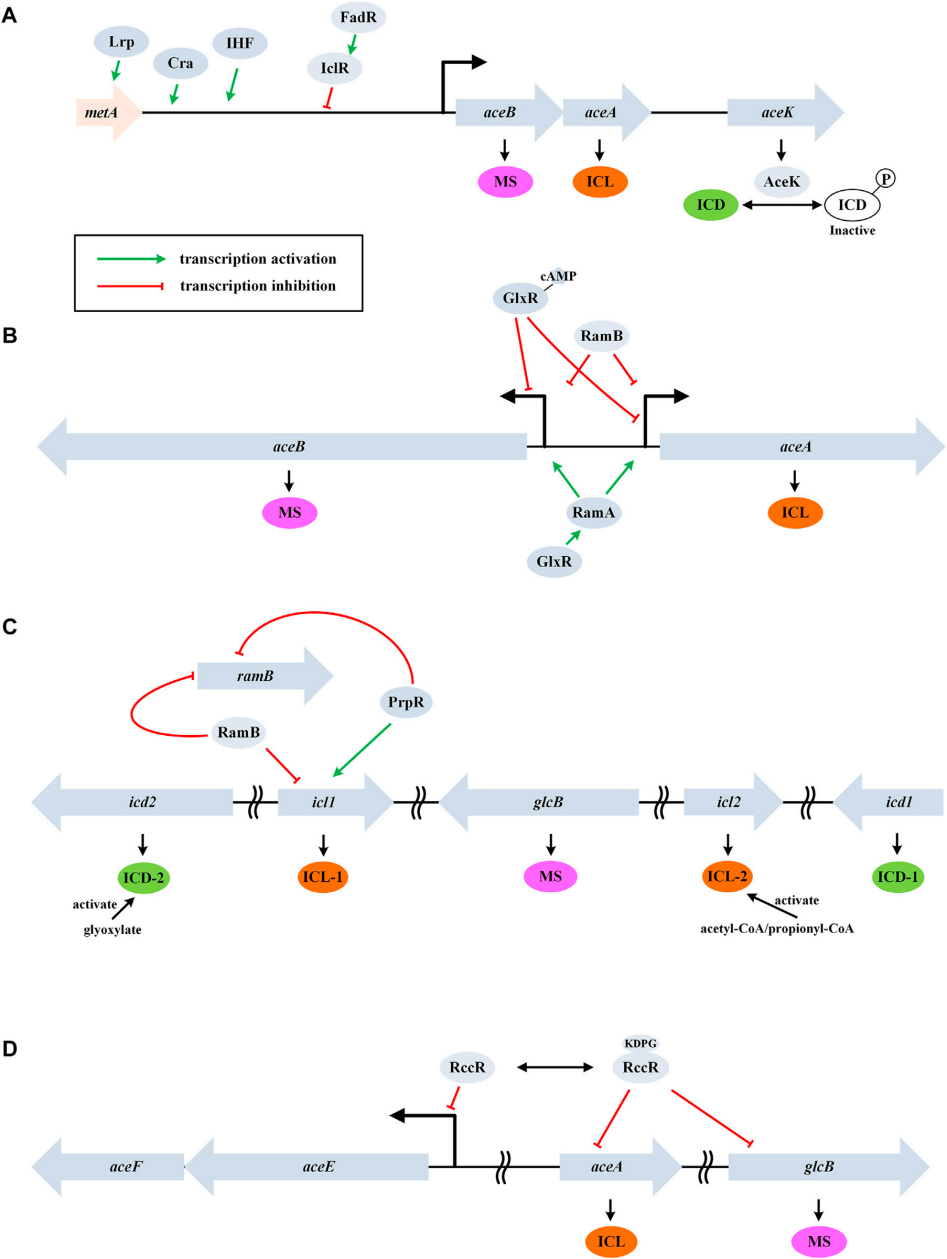
Control of the glyoxylate cycle in different bacteria. **(A)** GS regulation in the gram-negative *E. coli*. Genes *aceB*, *aceA*, and *aceK* form a tricistronic operon and AceK plays a critical role in controlling ICD activity through phosphorylation modification. **(B)** GS regulation in the gram-positive *C. glutamicum*. Genes *aceA* and *aceB* are transcribed divergently and a homologue of AceK is absent. **(C)** GS regulation in *M. tuberculosis*. This bacterium contains two isoforms of ICD and ICL. **(D)** GS regulation in *P. fluorescens*. RccR modulates pyruvate metabolism and GS *via* reciprocal regulation depending on the availability of KDPG. Genes *aceE* and *aceF* encode the pyruvate dehydrogenase components.

In *E. coli*, ICL is a tetramer encoded by *aceA*. Its affinity for isocitrate is much lower than that of the NADP-dependent isocitrate dehydrogenase (ICD, encoded by *icd*). As a result, the control of ICD activity plays an important role in determining the flux of GS (LaPorte et al., 1985). The activity of ICD is primarily controlled by a kinase/phosphatase called AceK (encoded by *aceK*) (Figure 2A). When bacteria are grown on acetate, about 75% of ICD is inactivated by phosphorylation, thus, more isocitrate will be directed through the glyoxylate cycle. The differential kinase/phosphatase activity of AceK is allosterically regulated by several metabolites (e.g., isocitrate, PEP, and ATP), although the precise mechanism is still not fully clear (Ogawa et al., 2007). It is worth noting that PEP also uncompetitively inhibits ICL.

AceK-dependent regulation of the flux through the glyoxylate cycle is a feature associated with only the gram-negative bacteria (this enzyme is absent in nearly all the gram-positive bacteria) (Yates et al., 2011). In addition, ICL is negatively regulated by acetylation in *E. coli* (Castano-Cerezo et al., 2014). On the other hand, MS (encoded by *aceB*) shows high affinities for the substrates glyoxylate and acetyl-CoA, and thus inhibition or activation of MS plays only a minor role in controlling the glyoxylate cycle (Anstrom et al., 2003).

In *E. coli*, the open reading frames (ORFs) of *aceB*, *aceA*, and *aceK* form a tricistronic operon (*aceBAK*), which is positively controlled by a pleiotropic transcriptional regulator Cra and the integration host factor (IHF) and negatively controlled by IclR (isocitrate lyase regulator) (Cozzone, 1998) (Figure 2A). The expression of the *aceBAK* operon can also be enhanced by Lrp, a transcriptional activator of the upstream *metA* gene (Kroner et al., 2019). Glyoxyalte and PEP stabilize the inactive dimeric state of IclR, while pyruvate stabilizes the active tetrameric form (Lorca et al., 2007). Expression of IclR is under self-inhibition and is activated by FadR (which represses β-oxidation and activates biosynthesis of fatty acids) (Gui et al., 1996). The coordination of fatty acid metabolism and the glyoxylate cycle may indicate acetyl-CoA as a signaling molecule.

### Control of GS in *Corynebacterium glutamicum*

Unlike the dimeric ICD enzyme from *E. coli*, the homologue (named IDH) is monomeric in *C. glutamicum*. The ORFs of *aceA* and *aceB* are transcribed divergently in *C. glutamicum* and a homologue of AceK is absent (Figure 2B). This organism does not encode an IclR homologue, while new types of transcription repressor RamB (regulator of acetate metabolism) and transcription activator RamA were characterized (Gerstmeir et al., 2004; Cramer et al., 2006). In the absence of acetate, RamB represses the expression of *aceA*, *aceB,* and *pta-ack* (encoding phosphotransacetylase and acetate kinase). The induction of glyoxylate cycle genes by acetate occurs independently of the presence or absence of glucose and other carbon source, which is different from *E. coli*.

Kim et al. reported a transcription repressor of *aceB* in *C. glutamicum*, which was designated as GlxR (Kim et al., 2004). The repression occurred in the presence of cAMP (e.g., when glucose medium was used). In addition, GlxR positively regulates *ramA* expression independent of the carbon source used (Toyoda et al., 2013) (Figure 2B). The glyoxylate cycle is also regulated through other mechanisms except for the transcriptional level. Maeda et al. found that RNase E/G (encoded by *rneG*) could cleave the *aceA* mRNA at the 3’ untranslated region, and the level of *aceA* mRNA was approximately 3-fold higher in the *rneG* mutant than in the wild type (Maeda and Wachi, 2012).

### Control of GS in *Mycobacterium tuberculosis* and *Pseudomonas fluorescens*

Bioinformatic studies showed that only microorganisms capable of aerobic metabolism possess the glyoxylate cycle, and the genetic context of related genes were diversified among bacterial genera, indicating more complex regulation (Ahn et al., 2016). For example, a totally different flux rheostat model was characterized in the *Mycobacterium* spp. (Figure 2C) and a reciprocal regulation model was characterized in *P. fluorescens* (Figure 2D). Although application of these strains in metabolic engineering was limited due to the pathogenicity, the mechanisms of GS regulation may be instructive.

*M. tuberculosis* contains two isoforms of ICD (dimeric Mtb ICD-1 and monomeric Mtb ICD-2), two isoforms of ICL (*E. coli*-like ICL-1 and a less-studied ICL-2), and lacks an AceK homologue (Munoz-Elias and McKinney, 2005). The *K*_*M*_s of ICL and ICD were comparable, in contrast with the situation in *E. coli* (Murima et al., 2016). The activity of ICD-2 (the primary isoform under physiological conditions) is stimulated by glyoxylate thereby decreasing the flux through GS. In this way, the flux balance between the TCA cycle and GS was achieved *via* a rheostat model. On glucose, RamB specifically represses the transcription of *icl1* and *ramB* itself (Micklinghoff et al., 2009). PrpR, a transcription factor involved in the methylcitrate pathway, can repress *ramB* expression and activate the expression of *icl1* (Masiewicz et al., 2012). A recent study showed that ICL-2 was markedly activated by acetyl-CoA and propionyl-CoA at high lipid concentrations (Bhusal et al., 2019).

At the posttranslational level, ICL-1 was found to be acetylated on three lysine residues (K322, K331, and K392). Acetylation at position 392 increased ICL activity, whereas acetylation of K322 reduced its activity (Bi et al., 2017). In ICD, residues K30 and K129 are acetylated by Rv2170, and this leads to a 30% reduction in the enzyme activity (Lee et al., 2017).

In *P. fluorescens*, RccR, a homologue of the Entner–Doudoroff pathway regulator HexR, plays the key role in the GS control *via* reciprocal regulation. In the presence of glucose, RccR binds 2-keto-3-deoxy-6-phosphogluconate (KDPG) and represses the expression of *aceA* and *glcB.* In the absence of KDPG, it represses the expression of *aceEF* genes. In this way, strain can modulate pyruvate metabolism and GS/gluconeogenesis in response to carbon source availability (Campilongo et al., 2017).

In several studies, unpredictable up/down-regulation of GS was resulted from the fermentation process control or genetic manipulation of the unrelated pathway due to interactional metabolism (Martinez et al., 2010; Wei et al., 2016; Borja et al., 2019). These results provided us new understanding of this pathway, yet were not readily applicable in the targeted metabolic engineering practice. These approaches will not be discussed in the present review.

## Production of organic acids

### Succinate production

Succinate has been considered as a potential chemical platform with a wide range of applications in food, pharmacy, chemical, and agriculture. Many microorganisms naturally accumulate succinate using the reductive TCA branch:

$$Glucose+2\;{CO}_{2}+2\;NADH=2\;Succinate+2\;{NAD}^{+}$$

**FIGURE 3:**
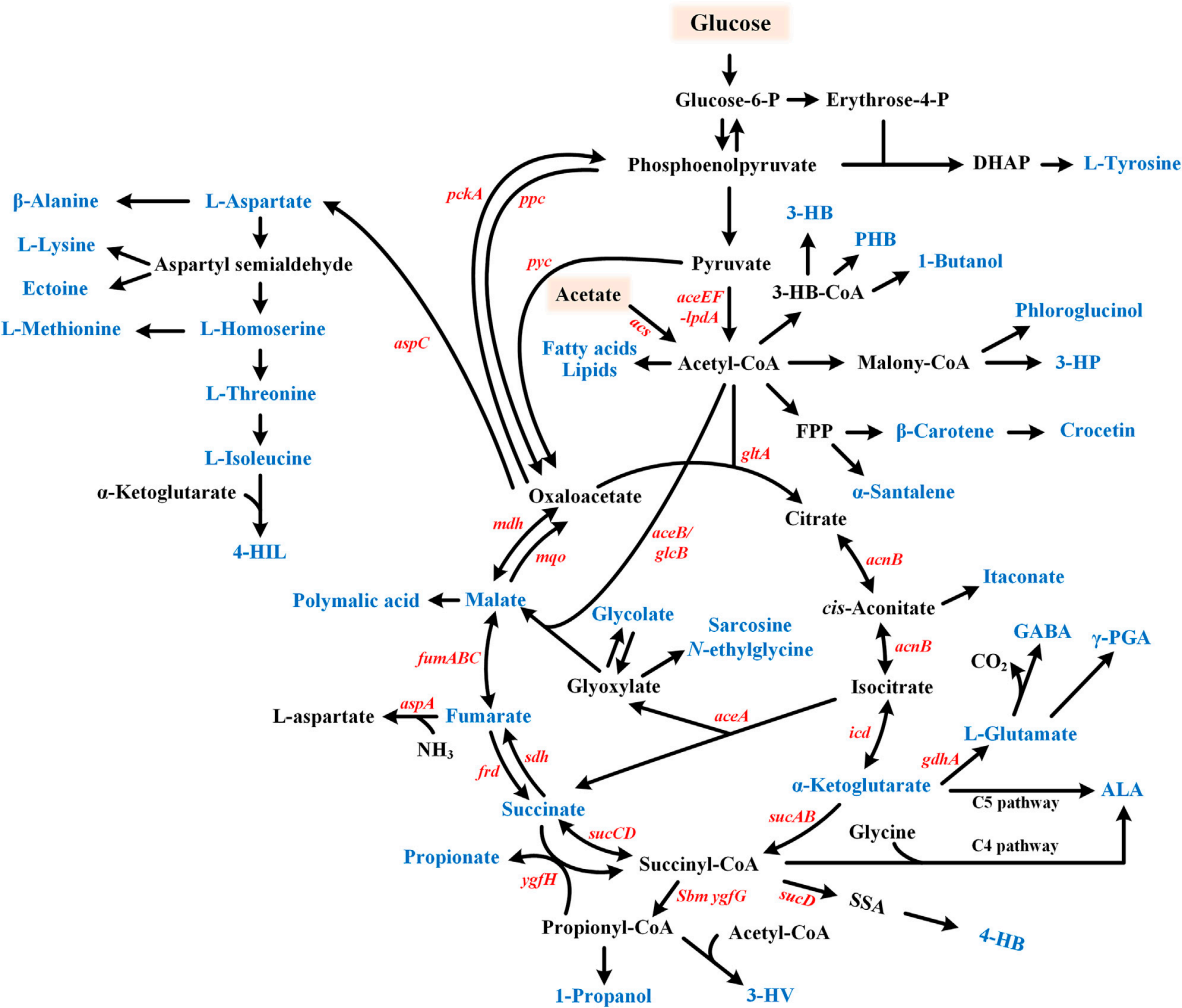
Metabolic pathway for the glyoxylate cycle involved chemicals production. Target products include organic acids, amino acids, fatty acid-related chemicals, etc. and are shown in blue. Glucose or acetate can be used as substrates. GABA, γ-aminobutyric acid; 4-HIL, 4-hydroxyisoleucine; ALA, 5-aminolevulinic acid; γ-PGA, Poly-γ-glutamic acid; 3-HB, 3-hydroxybutyrate; PHB, poly β-hydroxybutyric acid; 3-HP, 3-hydroxypropionate; 4-HB, 4-hydroxybutyrate; and 3-HV, 3-hydroxyvalerate.

However, the required NADH is typically generated by glucose heterofermentation, which reduces the theoretical yield to 1 mol/mol glucose (Figure 3). Ailen et al. developed a dual-route succinate production strategy through combining the reductive TCA part and the glyoxylate cycle (Sanchez et al., 2005). The lactate, ethanol, and acetate pathway were blocked to conserve NADH and acetyl-CoA. A heterologous pyruvate carboxylase (*pyc*) was overexpressed then to fix CO_2_ and increase the flux into the reductive TCA branch. Most importantly, conserved NADH and acetyl-CoA were channeled into the glyoxylate cycle by deletion of the repressor IclR. The engineered *E. coli* strain produced succinate at a yield of 1.6 mol/mol glucose under anaerobic conditions, which was close to the theoretical maximum (1.7 mol/mol glucose).

To achieve the theoretical maximum, the distribution of metabolic flux between carboxylation of PEP to OAA and decarboxylation of PEP to acetyl-CoA should be 5:2 (Vemuri et al., 2002). Skorokhodova et al. constitutively expressed the NAD^+^-reducing pyruvate dehydrogenase complex (*aceEF-lpdA*) to enhance the flux toward the glyoxylate cycle while supplying additional NADH at the same time (Skorokhodova et al., 2015). In an *E. coli* strain with blocked lactate, acetate, and ethonal pathways, they inactivated the pyruvate formate lyase (*pflB*) to drive acetyl-CoA formation solely by the overexpressed pyruvate dehydrogenase under anaerobic conditions. Further deletion of IclR with a concomitant heterologous pyruvate carboxylase (*pyc*) overexpression enabled a succinate yield of 1.69 mol/mol at tube-scale experiments with NaHCO_3_ addition.

Efforts were also put into the aerobic succinate production due to the faster cell growth and higher productivity compared with the anaerobic production. Recruitment of the glyoxylate cycle improved theoretical yield to 1.33 mol/mol from 1.0 mol/mol using the oxidative TCA part solely (Vuoristo et al., 2016). Under anaerobic conditions, ICD is many folds less active than under aerobic conditions (Chao et al., 1997). Also, considering its higher affinity to isocitrate compared with ICL, it is necessary to reduce or eliminate the expression of ICD to strengthen the glyoxylate pathway, and thus to improve the yield under aerobic conditions (Lin et al., 2005; Li et al., 2013; Li Y. et al., 2016). Currently, the titer or yield of succinate using aerobic fermentation is still low (Liu et al., 2022).

The glyoxylate cycle was also engineered in other microbes for succinate production except for *E. coli* (Zhu et al., 2014a; Cui et al., 2017; Babaei et al., 2019; Durall et al., 2021). Specially, Nguyen et al. integrated the GS enzymes from *E. coli* into a methanotroph *Methylomonas* sp. DH-1 (Nguyen et al., 2019). The GS pathway supplied intermediates for biomass synthesis in an *sdh* mutant and activited the serine cycle to provide more acetyl-CoA, which may have improved the cell growth and succinate production. Finally, 195 mg/L succinate, corresponding to 6.41% of the theoretical yield was produced from methane.

### Production of fumarate and malate

Succinate, fumarate, and malate can be interconverted but synthesis of fumarate and malate needs less reducing power compared with succinate under anaerobic conditions. In a study to produce fumarate, IclR was deleted to redirect carbon flux into the glyoxylate cycle (Song et al., 2013) (Figure 3). The phosphoenolpyruvate carboxylase (*ppc*) was overexpressed, then *arcA* (encoding a global transcriptional regulator) and *ptsG* (one of the phosphotransferase system genes) were deleted to reinforce the oxidative TCA cycle. Additionally, fumarate consumption was blocked through *fumABC* and *aspA* gene deletion. Finally, glucose uptake was increased by substituting the *galP* promoter. The engineered *E. coli* strain produced 28.2 g/L fumarate in 63 h under aerobic conditions (Song et al., 2013).

In another study, the decreased activity of ICD and activated GS was demonstrated to be effective to reduce acetate accumulation and increase the fumarate production. However, *aceBA* overexpression dramatically slowed cell gowth, which demonstrated the importance of flux balance between the GS and oxiditive TCA cycle (Li et al., 2014). Chen et al. engineered *E. coli* for fumarate production *via* the glyoxylate pathway (Chen et al., 2020). They explored the effect of pyruvate carboxylase (*pyc*), citrate synthase (*gltA*), aconitase (*acnB*), isocitrate lyase (*aceA*), and succinate dehydrogenase (*sdh*) overexpression on fumarate production, and found that pyruvate carboxylase and isocitrate lyase played crucial roles. The expression level of these two enzymes varied to balance the glyoxylate cycle and oxiditive TCA flux. Through pathway optimization, the fumarate titer was increased from 8.7 g/L to 16.2 g/L.

The glyoxylate pathway was also engineered for malate production in *E. coli* by the same research group (Gao et al., 2018). First, they constructed an *in vitro* malate production system containing five enzymes, which were, pyruvate carboxylase (PC), citrate synthase (CS), aconitase (ACN), isocitrate lyase (ICL), and malate synthase (MS), and explored the optimal level for each enzyme. Then, CRISPRi was employed for enzyme modular coordination *in vivo*. Through the moderate inhibition of PC and CS expression, and fixing the level of ACN: ICL: MS at 4: 5: 4, malate titer was increased by two folds with a yield of 0.85 mol/mol glucose. In addition, *iclR* and *arcA* were inactivated to enhance the flux toward glyoxylate cycle and oxiditive TCA cycle.

Trichez et al. engineered an *E. coli* strain for malate production through deletion of the *iclR* and *arcA* genes, block of malate consuming, and overexpression of malate-insensitive PEP carboxylase Ppc^K620S^ and NADH-insensitive citrate synthase GltA^R164L^. A malate yield of 0.82 mol/mol was obtained. The metabolic flux analysis indicated that the engineered strains had a very high flux over the glyoxylate shunt with almost no flux passing through the isocitrate dehydrogenase reaction (Trichez et al., 2018).

Liu et al. engineered L-malate production in *Aspergillus oryzae* (Liu et al., 2018). First, accumulation of intermediate pyruvate was decreased by overexpressing a pyruvate carboxylase from *Rhizopus oryzae* in the cytosol and mitochondria. Then, malate synthesis *via* the glyoxylate cycle in the mitochondria was enhanced by overexpression of isocitrate lyase and malate synthase. Interestingly, strengthening the oxidative TCA route diminished spore formation and malate production, while downregulation of the oxidative TCA cycle enhanced the L-malate titer by 10.7%, indicating that the TCA cycle route was not suitable for the malate production in this case. Finally, through the expression of a dicarboxylate carrier and modulation of the NADH/NAD^+^ ratio, 117.2 g/L L-malate was produced, with an L-malate yield of 0.9 g/g corn starch and a productivity of 1.17 g/L/h. Polymalic acid (PMA), a high added-value polyester composed of L-malic acid monomers, can be produced *via* the glyoxylate cycle naturally by *Aureobasidium pullulans*. A few studies engineered PMA production through enhancement of the glyoxylate cycle (Yang et al., 2018; Zeng et al., 2019).

### Production of itaconate

The glyoxylate cycle can also play a positive role in the production of itaconate, an excellent polymeric precursor derived from *cis*-aconitate (Figure 3). Through overexpression of pyruvate carboxylase, citrate synthase, aconitase, and the *cis*-aconitate decarboxylase (AT-CAD, *cad*) from *A. terreus*, flux toward the itaconate production was enhanced. Together with isocitrate dehydrogenase deletion, the titer of itaconate reached to 43 g/L in 32 h from glycerol under high aeration. Elimination of the glyoxylate shunt demonstrated harmful impact on the itaconate production, although this pathway pulled out the carbon flux *via* the isocitrate node. This may be due to its positive role in regeneration of oxaloacetate, which was important for itaconate synthesis in turn (Chang et al., 2017).

In another study, an acid-tolerant *E. coli* strain was engineered for itaconate production from acetate (Noh et al., 2018). By overexpression of the *cis*-aconitate decarboxylase, only 0.13 g/L itaconic acid was produced because of low acetate uptake. Then, acetate assimilation was enhanced through overexpression of the acetyl-CoA synthetase and activation of the glyoxylate cycle. The high ICL activity was proved to be benificial for the itaconate production. Together with *gltA* overexpression, 3.57 g/L itaconic acid (16.1% of theoretical maximum yield) was produced from acetate.

### Production of glycolate

Glycolate, or glycolic acid (GA) is a small two-carbon α-hydroxy acid used in multiple daily applications (Figure 3). There are no known natural microbial pathways to directly produce GA from relatively cheap feedstock, yet this chemical can be produced *via* several synthetic pathways including the modified GS pathway (Salusjärvi et al., 2019). In an attempt to produce GA using *E. coli*, flux toward the glyoxylate cycle was enhanced through the overexpression of ICL and deletion of acetate forming pathways, *icd*, *arcA*, and *iclR*. GA synthesis was further enhanced by glyoxylate reductase (*ycdW*) overexpression. Consumption of glyoxylate and GA was blocked through inactivation of MS, glyoxylate carboligase (*gcl*), 2-keto 3-deoxygluconate 6-phosphate aldolase (*eda*), glycolate oxidase (*glcDEFGB*) and glycolaldehyde dehydrogenase (*aldA*). To increase the availability of NADPH, which is needed by YcdW, *edd* encoding the 6-phosphogluconate dehydratase was deleted. Finally, 52.2 g/L GA was produced from glucose (Dischert and Soucaille, 2015).

Sometimes complete block of the TCA cycle through *icd* deletion may cause growth retardation. Deng et al. tried to weaken the flux to α-ketoglutarate (α-KG) through overexpression of AceK, which could repress the ICD activity. In this way, the ICD activity was decreased by 83.03%, but poor growth of strain was incurred concomitantly. Then, adaptive evolution was performed to increase the growth rate. Nevertheless, the evolved strain still grew much more slowly than the wild type, which suggested the importance of balance between the TCA cycle and GS reactions. In fed-batch fermentation, the engineered strain produced 56.4 g/L GA (Deng et al., 2015). Subsequently, Deng et al. further improved the producer strain (Deng et al., 2018). Citrate synthase was overexpressed in addition to ICL, YcdW, and AceK. Lactate dehydrogenase (*ldhA*), which competed for the carbon flux to glycolate and AldA involved in GA consumption were deleted. The following optimization of fermentation, 65.5 g/L GA was produced with a yield of 0.765 g/g glucose (90.0% of the theoretical yield).

Pereira et al. engineered an *E. coli* strain to produce GA from xylose, the most abundant pentose (Pereira et al., 2016). Through introduction of the exogenetic D-tagatose epimerase, 44.0 g/L GA was produced *via* the D-ribulose-1-phosphate pathway. Bu combining the D-ribulose-1-phosphate pathway and glyoxylate cycle, the yield was increased from 0.44 to 0.62 g/g xylose (theoretical yield 1 g/g), although the titer did nott improve obviously. Cam et al. engineered GA production in *E. coli* from xylose *via* a synthetic xylulose-1 phosphate (X1P) pathway (Cam et al., 2016). In this pathway, D-xylose was converted to glycolaldehyde and DHAP, both of which can be converted to GA with a theoretical yield higher than 20% *via* the glyoxylate shunt alone. Simultaneous operation of the glyoxylate and X1P pathways enabled a yield of 0.63 g/g, when growing on the glucose/xylose mixture (Alkim et al., 2016).It is worth noting that when produced from xylose *via* the Dahms pathway, the yield of GA was only 0.5 g/g xylose (Cabulong et al., 2021).

Li et al. engineered GA production in *E. coli* from acetate *via* the glyoxylate cycle (Li et al., 2019). The glyoxylate bypass was reinforced by overexpression of ICL and AceK. YcdW was overexpressed to convert glyoxylate to glycolate. MS, glyoxylate carboligase, and glycolate oxidase were inactivated to prevent loss of glyoxylate and glycolate. To strengthen the TCA cycle and acetate utilization, citrate synthase, phosphotransacetylase, and acetate kinase (*ackA*) were co-overexpressed. As a result, the glycolate titer was improved to 2.75 g/L with the pH control in shake flasks. The GA production pathway can be integrated with other metabolic routes to produce value-added chemicals including 3-hydroxy-γ-butyrolactone (3HBL) and GA-containing polymer directly from glucose or/and xylose (Dhamankar et al., 2014; Choi S. Y. et al., 2016; Li Z.-J. et al., 2016; Li Z.-J. et al., 2017). In addition, the glyoxylate cycle was also engineered to produce GA in microbes other than *E. coli*, although the titer was relatively low (Koivistoinen et al., 2013; Zahoor et al., 2014; Lee et al., 2020).

## Production of amino acids and derivatives

### Production of L-aspartate family amino acids

Several amino acids and their derivatives are synthesized from the GS and TCA cycle intermediates, including OAA, α-KG and succinyl-CoA (Figure 3). L-Aspartate family amino acids (AFAAs) refer to amino acids synthesized from L-aspartate such as L-lysine, L-methionine, L-threonine, and L-isoleucine. Because L-aspartate is directly synthesized from OAA, the OAA supply has been considered as a bottleneck for the production of AFAAs (Li Y. et al., 2017). In one study, the OAA pool was increased to improve L-homoserine production in *E. coli* (Liu et al., 2020). First, IclR was deleted to derepress the glyoxylate cycle. Then, citrate synthase (*gltA*) was deleted to conserve OAA. Subsequently, pyruvate kinase (*pykA* and *pykF*) was deleted to drive more anaplerotic flux into OAA. Together with disrupting the competitive and degradative pathways, 35.8 g/L L-homoserine was produced in the fed-batch fermentation. Conversely, simultaneous activation of the glyoxylate cycle and overexpression of *gltA* (rather than weakening its expression) improved AFAAs production in some other studies, which suggested the complexity of metabolism (Flores et al., 2005; Zhu et al., 2019). Sometimes, the glyoxylate cycle could be activated through attenuating the isocitrate dehydrogenase activity, which also resulted in replenishment of OAA (Schwentner et al., 2018).

In another study, L-threonine, which can be synthesized from L-homoserine, was produced in *E. coli* (Zhao et al., 2018). To conserve precursor OAA, the gene *iclR* was deleted, and the native promoter of the *aceBA* operon was replaced by the strong *trc* promoter in the chromosome. Then, the L-threonine biosynthesis pathway was overexpressed *via* replacing the native promoter of *aspC* (aspartate aminotransferase) by the *trc* promoter in the chromosome and plasmid overexpression of thrA* (a mutated *thrA*), *thrB*, *thrC,* and *asd* (encoding aspartate kinase I, homoserine kinase, threonine synthase and aspartate semialdehyde dehydrogenase, respectively). The final strain TWF006/pFW01-thrA*BC-asd produced 15.85 g/l L-threonine with a yield of 0.53 g/g glucose. In a subsequent study, the same group found that increase of the acetyl-CoA pool positively affected L-threonine production (Yang et al., 2019). The acetyl-CoA pool was increased through: deletion of fatty acid degradation/synthesis regulator FadR and FabR; overexpression of acetyl-CoA synthetase (*acs*) to convert acetate into acetyl-CoA; and overexpression of *fadBA* to facilitate fatty acid degradation. The fatty acid degradation and L-threonine biosynthesis pathway were coupled *via* the glyoxylate shunt, and 103.89 g/l L-threonine was produced after 48-h fed-batch fermentation.

Ectoine is a protective agent and stabilizer which can be synthesized from L-Aspartate. Ning et al. introduced the synthesis pathway of ectoine (encoded by the *ectABC* gene cluster from *Halomonas elongata*) into *E. coli* to produce this valuable chemical (Ning et al., 2016). To increase the OAA pool, *iclR* was deleted to enhance the glyoxylate shunt and the expression level of *ppc* (encoding the phosphoenolpyruvate carboxylase) was improved through promoter change. Then, the synthetic pathway of L-lysine and L-threonine was blocked, and a feedback resistant LysC from *C. glutamicum* (encoding the aspartate kinase) was introduced to enhance the flux to ectoine. Together with the overexpression of *ectABC*, 25.1 g/L ectoine was produced by fed-batch fermentation. Increase of the OAA pool *via* deletion of *iclR* was also proved to be effective in a study to produce β-alanine, another AFAA in *E. coli* (Wang et al., 2021). However, further enhancement of GS genes through promoter change did not improve β-alanine production, suggesting the importance of flux balance.

### Production of α-ketoglutarate-sourced amino acids

α-KG is the precursor of numerous amino acids, such as L-glutamine, L-glutamate, and L-proline (Figure 3). In a *C. glutamicum* L-glutamate producer strain, glyoxylate pathway was blocked by knocking out *aceA* to conserve the isocitrate pool, and glutamate synthesis was blocked by knocking out *gdh* (encoding glutamate dehydrogenase). As a result, α-KG production was increased by 16-fold to 12.4 g/L in flask culture and 47.5 g/L in 5-L fermentor (Jo et al., 2012). In another study to produce Poly-γ-glutamic acid (γ-PGA) using *Bacillus licheniformis*, the glyoxylate cycle was also reduced to improve the titer (Li et al., 2021). The expression level of *aceBA* was varied through promoter modulation, and with a weak promoter P_*bacA*_, the activity of ICL was decreased by 41.51%, which resulted in 23.24% increase in γ-PGA yield. In addition, pyruvate dehydrogenase and citrate synthase were overexpressed to strengthen the flux into the TCA cycle, and pyruvate formate-lyase was deleted to conserve pyruvate. Finally, γ-PGA titer was enhanced to 12.02 g/L.

In biofermentation, it is essential to address the conflict between cell growth and target chemical production, as the overexpressed production pathways often lead to metabolic burden for the cell. In a study to produce γ-aminobutyric acid (GABA), a genetic switch was designed to balance the GABA production and bacterial cell growth (Soma et al., 2017). The cell growth was controlled by conditional interruption of GS and the TCA cycle. In the cell growth mode, α-ketoglutarate decarboxylase (*sucA*) of the TCA cycle and ICL (*aceA*) of the glyoxylate cycle were actively expressed, while turned off in the GABA production mode. On the contrary, pyruvate carboxylase (*pyc*), glutamate dehydrogenase (*gdhA*), glutamate decarboxylase (*gadB*), and the GABA transporter (*gadC*) involved in GABA synthesis were turned on only during the production mode. This strategy balanced the competition for isocitrate and α-KG between cell growth and GABA production, resulting in a 3-fold improvement in the total GABA production titer and yield.

In another study to produce 4-hydroxyisoleucine (4-HIL), a potential medicine for diabetes, the glyoxylate cycle was blocked (by deleting *aceA*) to increase one of the substrates α-KG (Shi et al., 2019). 4-HIL was synthesized from L-isoleucine and α-KG under the activity of L-isoleucine dioxygenase (IDO). The deletion of *aceA* increased the concentration of both α-KG and L-isoleucine, resulting in an 18.9% increase in the titer of 4-HIL. Then, *ido3* (another IDO encoding gene) and *mqo* (encoding malate:quinone oxidoreductase) were coexpressed with *ido* to draw more flux into the Ile and 4-HIL biosynthetic pathways, resulting in another 31.8% increase of the titer. Further expression of the *Vitreoscilla* hemoglobin (*vgb*) and optimization of the fermentation medium led to a final 4-HIL titer of 17.2 g/L.

5-Aminolevulinic acid (ALA) is an α-KG derived non-proteinogenic amino acid with multiple applications in medical, agricultural, and food industries. There are two distinct routes to produce this chemical biologically, the C_5_ pathway and the C_4_ pathway. In the C_5_ pathway, ALA is synthesized from α-KG. To conserve α-KG, the TCA cycle was blocked by deletion of *sucA* (encoding α-ketoglutarate decarboxylase) (Noh et al., 2017). Although the specific ALA production was increased obviously, both ALA titer and cell biomass were reduced probably due to the insufficient production of energy and building blocks. Overexpression of *gltA* encoding citrate synthase only marginally improved cell biomass. Next, the glyoxylate cycle was finely tuned through modulating the promoter of *aceA*. Under the optimal strength, cell biomass and ALA production were increased by 4.45-fold and 2.93-fold, respectively, as compared to the parental strain. After fermentation optimization, high productivity (0.19 g/L/h) and yield (0.28 g/g) for ALA production from glucose as a sole carbon source was achieved.

In the C_4_ pathway, ALA was synthesized from succinyl-CoA and glycine. To reduce the conversion of ALA to downstream tetrapyrrole/porphyrin, Miscevic et al. applied CRISPRi to repress *hemB* expression (Miscevic et al., 2021b). Under microaerobic condition, succinyl-CoA is mainly derived from reductive the TCA cycle and the glyoxylate cycle. The authors inactivated *iclR* to deregulate the glyoxylate shunt while deleting *sdhA* simultaneously to further redirect the carbon flux toward ALA biosynthesis. Finally, 6.93 g/L ALA was produced from 30 g/L glycerol in *E. coli*. In another study, an exogenous glyoxylate transaminase from the human, which enabled autogenous synthesis of glycine from glyoxylate, was introduced into *E. coli* to produce ALA using glucose as the sole carbon source (Ren et al., 2018). The transaminase used alanine as the amino donor, with ATP, PLP, and CoA as cofactors. To increase the supply of glyoxylate, *aceA* was overexpressed. As a proof-of-concept, 521 mg/L 5-ALA was produced in 18 h of fermentation.

### Production of tyrosine and *N*-alkylated glyoxylate

Tyrosine is an aromatic amino acid, which can be utilized in many aspects. In a study to engineer tyrosine production from acetate in *E. coli*, precise tuning of the glyoxylate cycle was proved to be vital (Jo et al., 2019). In this case, the two important intermediates for tyrosine synthesis, erythrose-4-phosphate and PEP were both derived from OAA. As acetate was the sole carbon source, it was critical to balance the glyoxylate cycle and TCA cycle, in other words, to balance precursor supply and generation of ATP and NADH. The glyoxylate cycle was precisely controlled by modulating the promoter of *aceA*. As a result, the best engineered strain produced 0.70 g/L tyrosine.

Glyoxylate can be *N*-alkylated by monomethylamine or monoethylamine, forming sarcosine and *N*-ethylglycine, respectively, under the activity of imine reductases DpkA (Mindt et al., 2019b). To increase glyoxylate supply, *aceB* was deleted and the activity of isocitrate dehydrogenase was reduced by changing the preferred translational start codon ATG to GTG. To further activate the glyoxylate cycle, acetate was added into the culture media. Together with other strategies including optimization of carbon source species, the amount and addition time of monomethylamine and acetate, 8.7 g/L sarcosine was produced from xylose and potassium acetate blends using the engineered *C. glutamicum*. Subsequently, DpkA was mutated, which accelerated the production of sarcosine (Mindt et al., 2019a). Using this mutant, production of *N*-ethylglycine from xylose and monoethylamine or from rice straw hydrolysate was demonstrated.

## Production of fatty acid-related chemicals and farnesyl diphosphate-derived bioactive compounds

### Production of acetyl-CoA derivatives

Synthesis of fatty acids originates from acetyl-CoA in microbial cells. Acetyl-CoA lies at the entrance into the TCA cycle, and is closely related with the glyoxylate cycle. Many polyhydroxyalkanoate monomers and several bioactive compounds can be synthesized from acetyl-CoA, as shown in Figure 3. On the other hand, propionate and 4-hydroxybutyrate (4-HB) can be synthesized from succinyl-CoA, another intermediate strongly linked with GS. Thus, the glyoxylate cycle was engineered to improve production of related chemicals in a few studies.

Nitta et al. engineered an *E. coli* strain to produce 1-butanol, a bulk chemical and promising biofuel (Nitta et al., 2019). All native fermentation pathways were blocked (through deletion of *ldhA*, *adhE,* and *frdBC*) and the strain relied on 1-butanol formation as the sole electron sink to regenerate NAD^+^. 1-Butanol was produced using a heterologous Clostridial CoA-dependent pathway. Then, formate dehydrogenase (*fdh*) from *Candida boidinii* was introduced to supply more NADH. Also, phosphate acetyltransferase (*pta*) was deleted to prevent formation of by-product acetate. In addition, the expression of AdhE2, the key enzyme for 1-butanol synthesis was optimized, which resulted in a 1-butanol titer of 18.3 g/L. However, considerable amounts of acetate accumulated (Ohtake et al., 2017). Subsequent metabolome analysis revealed increased accumulation of glyoxylate and acetyl-P with a decrease of α-KG and glutamate in the engineered strain, compared with the base strain. By knocking out *aceA*, acetate production decreased by 72% and the TCA cycle metabolites (including α-KG and glutamate) increased, which resulted in a 10% increase of the cell growth. Finally, 1-butanol production was improved by 39% (Nitta et al., 2019).

In another study, the expression of the glyoxylate cycle was enhanced by knocking out *iclR* to overcome acetate overflow and improve the production of two acetyl-CoA derived chemicals, phloroglucinol (PG) and 3-hydroxypropionate (3-HP) in *E. coli* BL21 (Liu et al., 2017). Acetate accumulation inhibits protein synthesis and depletes the acetyl-CoA pool. Deletion of *iclR* decreased acetate formation and improved the glucose utilization efficiency, which could reduce the production cost. At the same time, the metabolic flux from acetate and PEP to acetyl-CoA was enhanced, resulting in a more than 2-fold increase in the production of PG or 3HP. In a previous study, deletion of *arcA*, the redox regulator known to repress the TCA cycle and glyoxylate cycle, demonstrated similar effects on cell physiology and production of acetyl-CoA derived chemicals (Liu et al., 2016). However, the *arcA* and *iclR* double mutant showed no better results for PG and the 3HP production than *arcA* or *iclR* single mutant and the mechanisms were unclear. In another study, to produce 3-HP in *E. coli* from acetate, substrate assimilation was enhanced by overexpressing *acs* and the GS was activated by deleting *iclR*. As a result, acetate uptake and cell biomass synthesis were enhanced significantly, and 3-HP production was improved by 2.54-fold (Lee et al., 2018).

To balance the cell growth and 3-HP synthesis, a two-stage strategy was adopted by Lama et al., whereby glucose is used for the cell growth and acetate for 3-HP synthesis (Lama et al., 2021). To increase biomass yield on glucose, pathways for synthesis of by-products lactic acid, ethanol, and acetic acid were removed. Then, the effects of GS and the gluconeogenesis pathways on the cell growth and 3-HP formation during the production stage were studied. As a result, block of gluconeogenesis or GS was detrimental for 3-HP production, while theactivation of GS (*via* deletion of *iclR*) improved the titer of 3-HP. Using fed-batch fermentation, 7.3 g/L 3-HP was produced with a yield of 0.26 mol/mol acetate. Notably, the yield was still low compared with the theoretical maximum of 0.5 mol/mol acetate, which may reflect the operational cost of GS and subsequent gluconeogenesis.

3-HP biosynthesis was also pursued in non-model microorganisms. Using *P. denitrificans* as the producer, activation of GS by deleting *iclR* or enhancement of the oxidative TCA branch by *aceK* deletion did not improve 3-HP significantly, when a similar two-stage production strategy was adopted (Zhou et al., 2020). Lai et al. deleted the fatty acid degradation repressor FadR to enhance acetic acid utilization, which also activated the GS and improved 3-HP production. Using whole-cell biocatalysis, 15.8 g/L and 11.2 g/L of 3-HP was produced from acetate or syngas-derived acetate, respectively (Lai et al., 2021). Sometimes, when a target chemical not so closely-related was produced, the impact of GS would be complicated (Lin et al., 2013; Zhang et al., 2019).

To enhance production of FPP-derived bioactive compounds, the key point was usually the acetyl-CoA level. Citric acid synthase (CIT2 in peroxidase body) or malic acid synthase (MLS1 in the cytoplasm) was knocked out in a study to enhance the supply of precursor acetyl-CoA for production of crocetin, a potential drug in *Saccharomyces cerevisiae* (Song et al., 2020). The CIT2 mutant achieved 50% improvement on the total acetyl-CoA content and crocetin production, compared with the parent strain. The MLS1 mutant demonstrated similar effects, but to a lesser degree. Followed by suited fusion expression of key enzymes CrtZ and CCD2 and medium optimization, 12.43 mg/L crocetin was produced in fed-batch fermentation. Deletion of CIT2 and/or MLS1 also proved beneficial to increase the acetyl-CoA level in other studies. In a study to produce α-santalene (an acetyl-CoA derived sesquiterpene) in an engineered yeast strain, deletion of CIT2 or MLS1 improved the titer by 36% and 127%, respectively (Chen et al., 2013). Using CIT2 or MLS1 mutant, the titer of butanol was improved to 14.0 and 16.3 mg/l, respectively in another study (compared with 10.3 mg/L in the parent yeast strain) (Krivoruchko et al., 2013). However, block of the glyoxylate cycle through deletion of CIT2 or MLS1 compromised cell growth of a PHB-producing yeast strain and resulted in accumulation of acetate and a decrease in PHB content (Kocharin et al., 2012).

### Production of succinyl-CoA derivatives

Under the anaerobic condition, succinate was mainly derived from the reductive TCA branch. However, the glyoxylate cycle could play an important role under the aerobic/microaerobic condition. Thus, effects of GS on the succinyl-CoA level were quite DO-dependent. In a study to produce propionate using the sleeping beauty mutase (Sbm) pathway in *E. coli*, the oxidative TCA cycle was blocked *via* deletion of *sdhA* and the glyoxylate cycle was deregulated by *iclR* deletion to improve the succinyl-CoA level. Through this modification, propionate titer was increased to 3.68 g/L from 1.1 g/L. Finally, 30.9 g/L of propionate was produced with an overall yield of 49.7% under optimized aeration condition using fed-batch fermentation (Miscevic et al., 2020b). Subsequently, 3-hydroxyvalerate was produced through condensation of propionyl-CoA and acetyl-CoA *via* a heterologous pathway using glycerol as the carbon source (Miscevic et al., 2020a; Miscevic et al., 2020c). The effects of aeration condition on activity of GS, and thus, flux distribution into the Sbm pathway were further demonstrated during production of poly (3-hydroxybutyrate-co-3-hydroxyvalerate) (Miscevic et al., 2021a).

4-HB is another chemical derived from succinyl-CoA that can be converted into various industrial chemicals and polymers. Producer strains of *E. coli* engineered for both aerobic and microaerobic conditions were constructed by Choi et al. based on a genome-scale metabolic model (Choi S. et al., 2016). Under the aerobic condition, the oxidative TCA branch and GS were proved to be essential for the supply of precursor succinate and production of 4-HB. Deletion of *sdhAB* and *iclR* was efficient to increase the titer of 4-HB. However, neither enhancement of GS (*via* deletion of *iclR*) nor block of GS (*via* deletion of *aceBA*) were effective in enhancing 4-HB production under the microaerobic condition, and there seemed to exist an optimal expression level of GS. In addition, glycerol was proved to be effective only for the microaerobic producer. Finally, 103.4 g/L of 4-HB was produced by microaerobic fed-batch fermentation from glycerol.

Santala et al. engineered wax esters production in *Acinetobacter baylyi* ADP1 from acetate and gluconate (Santala et al., 2021). By deleting *aceA*, GS was blocked and consumed acetate was dedicated to wax esters biosynthesis. On the other hand, gluconate was used for the synthesis of cofactors and biomass precursors. This design overcame the trade-offs between biomass and product production, an issue frequently encountered in bioengineering. Through optimization of gluconate feed rate and enhancement of the wax esters pathway, wax esters content, titer and productivity were improved significantly and a yield of 18% C/C-total-substrates was achieved.

## Concluding remarks and outlook

As outlined in this review, the biochemistry and regulation of glyoxylate cycle was demonstrated in two industrial microorganisms (*E. coli* and *C. glutamicum*) and two potential pathogenic microorganisms (*M. tuberculosis* and *P. fluorescens*). Although applications in biosynthesis by *M. tuberculosis* and *P. fluorescens* are limited due to the pathogenicity, the GS control mechanisms may be instructive. Production of target chemicals, divided into several categories, including organic acids, amino acids and fatty acid-related chemicals were also briefly introduced.

Currently, metabolic engineering studies are focused on a small number of model microorganisms, especially *E. coli*. Thus, other potential producer strains with better robust traits, production capacity and feedstock compatibility may have been missed (Zeng, 2019). Considering the deep involvement of glyoxylate cycle in the synthesis of so many chemicals, clear interpretation of the glyoxylate metabolism in other industry relevant microorganisms will undoubtedly facilitate chemical bioproduction in the future (such as yeast, cyanobacteria, *streptomyces* and Clostridia (Chen et al., 2012; Huang et al., 2015; Zhang and Bryant, 2015)). On the other hand, studies on more detailed effects of exogenously introduced the GS pathway on the physiology and production performance of the producers are encouraged (Kabisch et al., 2013; Schada von Borzyskowski et al., 2018; Shimizu et al., 2019). In addition, protein engineering studies of the GS pathway, particularly for biosynthesis purpose are barren to our knowledge.

Metabolism of substrates which could be facilitated by GS are not just limited to acetate, glycerol or fatty acids. Recently, Mainguet et al. constructed a reverse GS which could convert C_4_ carboxylates into two molecules of acetyl-CoA without loss of CO_2_ (Mainguet et al., 2013). Heterologous enzymes malate thiokinase, malyl-CoA lyase and ATP-citrate lyase were utilized to drive the thermodynamically unfavorable steps, namely the conversion of malate to glyoxylate and acetyl-CoA, and conversion of citrate to OAA and acetyl-CoA at the expense of ATP. When adapted and integrated with central metabolism, conversion of C1 or CO_2_ carbon source to acetyl-CoA can be realized (Yu et al., 2018; Yu and Liao, 2018). Although no practical production use of this pathway has been implemented, this non-native route holds the potential for producing various acetyl-CoA derived chemicals, including alcohols, fatty acids, and isoprenoids in the future.

**FIGURE 4:**
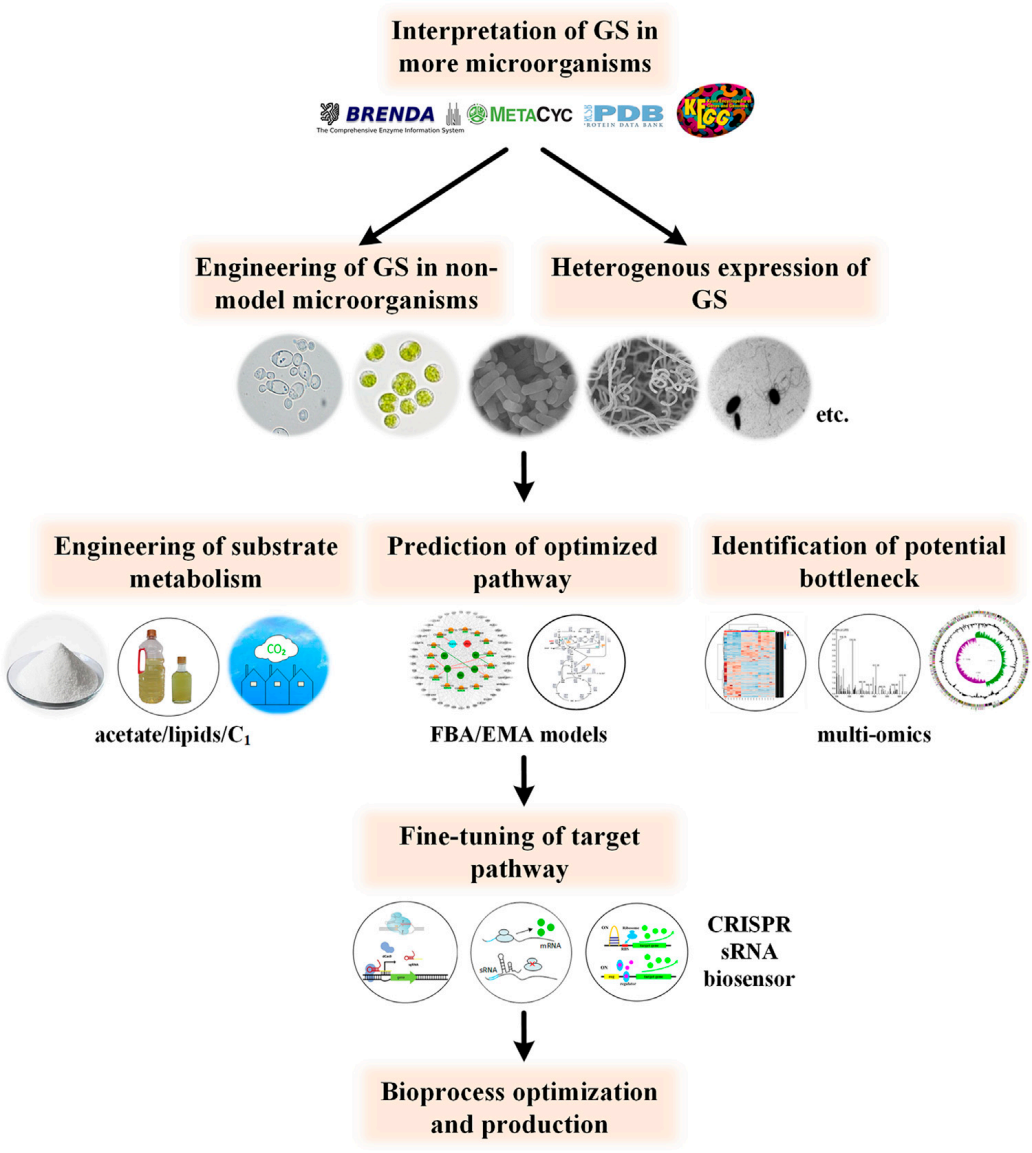
Perspective for future studies concerning GS engineering. Further understanding of GS metabolism in more potential producer microorganisms and more precise modulation of related pathways will promote the bioproduction process.

Another challenge is the balance of the cell growth and product biosynthesis. This may be overcome in two steps, that is,, accurate prediction of the appropriate GS strength and fine-tuning of its expression level accordingly. Prediction of the optimized metabolic flux can be promoted by multi-omics and more precise genome-scale metabolic models, such as flux balance analysis (FBA)-based models, elementary mode analysis-based ones and kinetic models (Srirangan et al., 2013; Machado and Herrgård, 2015). Then, fine-tuning of the GS pathway could be realized through CRISPRi, CRISPRa, sRNA, or dynamic control (Choi et al., 2019). The outline for future studies is shown in Figure 4. With the development of new techniques and expansion of the online database, the product portfolio and substrate spectrum can be further expanded in the future.

## Abbreviations

AFAA, L-aspartate family amino acid; ALA, 5-aminolevulinic acid; GS, glyoxylate shunt; GABA, γ-aminobutyric acid; 4-HIL, 4-hydroxyisoleucine; 3-HP, 3-hydroxypropionate; 4-HB, 4-hydroxybutyric acid; ICL, isocitrate lyase; ICD, isocitrate dehydrogenase; IclR, isocitrate lyase regulator; α-KG, α-ketoglutarate; MS, malate synthase; OAA, oxaloacetate; PEP, phosphoenolpyruvate.
